# Conscientious objection to intentional killing: an argument for toleration

**DOI:** 10.1186/s12910-018-0323-0

**Published:** 2018-10-19

**Authors:** Bjørn K. Myskja, Morten Magelssen

**Affiliations:** 1 Department of Philosophy and Religious Studies, Norwegian University of Science and Technology - NTNU, NO-7491 Trondheim, Norway; 2 Centre for Medical Ethics, Institute of Health and Society, University of Oslo, NO-0318 Oslo, Norway

**Keywords:** Conscience, Abortion, Assisted dying, Pluralism, Tolerance, Democracy

## Abstract

**Background:**

In the debate on conscientious objection in healthcare, proponents of conscience rights often point to the imperative to protect the health professional’s moral integrity. Their opponents hold that the moral integrity argument alone can at most justify accommodation of conscientious objectors as a “moral courtesy”, as the argument is insufficient to establish a general moral right to accommodation, let alone a legal right.

**Main text:**

This text draws on political philosophy in order to argue for a legal right to accommodation. The moral integrity arguments should be supplemented by the requirement to protect minority rights in liberal democracies. Citizens have a right to live in accordance with their fundamental moral convictions, and a right to equal access to employment. However, this right should not be unconditional, as that would unduly infringe on the rights of other citizens. The right must be limited to cases where the moral basis is more fundamental in a sense that all reasonable citizens in a liberal democracy should accept, such as the constitutive role of the inviolability of human life in liberal democracies.

**Conclusion:**

There should be a legal, yet circumscribed, right to accommodation for conscientious objectors refusing to provide healthcare services that they reasonably consider to involve the intentional killing of a human being.

## Background

Arguments for a right to conscientious objection for health professionals usually emphasize the value of moral integrity [1] or, less often, the related idea of the special nature of healthcare professions as vocations [2]. Moral integrity is valuable to the person who possesses it, and society benefits from citizens cultivating such integrity. The need to protect health professionals’ moral integrity is a prima facie reason for not requiring them to act contrary to their conscience. However, when considered on its own, the “moral integrity argument” has been strongly criticised. Such lines of argument taken in isolation, it is argued, will at most support a moral duty for others to accommodate as “a moral courtesy” [3], on the condition that *nothing of equal or greater moral significance is affected* [4]. As the employee has voluntarily chosen the position and can protect her integrity by resigning, the employer’s moral duty to accommodate will be outweighed when there are significant burdens to patients, colleagues or employer.

Although there are many instances of conscientious objection that may be accommodated without morally significant negative consequences, it is difficult to imagine any *general* moral right to exemption from professional duties that never will have singular cases where there are morally significant consequences. For the patient, such consequences may consist in four kinds of harm: delayed or restricted access to services; increased expenses; lack of important information; and the communication of moral disapproval of the patient’s choices or lifestyle [5]. Such consequences may be prevented by colleagues performing the functions for the objector, but this may come at some cost to these colleagues, such as increased workload or reduced diversity of work tasks. Accommodation may also incur costs for employers and institutions. The implication is that although there are good reasons to have policies of accommodations as conditional moral courtesies, there cannot be a moral *right* to exemption based solely on the interest of protecting moral or vocational integrity. In other words, although the conscientious objector’s moral integrity being at stake is a *necessary* reason for tolerating their refusal, it is not a *sufficient* reason for such toleration being a moral or political requirement.

This conclusion is supported further by worries that integrity or vocation based arguments are ill-suited to discriminate between claims for accommodation that *ought to be granted*, and claims that *ought to be rejected*. If such arguments cannot provide the necessary help in assessing the moral value of different claims for accommodation, we would be forced to accept a wide range of exemptions in order to avoid the charge of discrimination. As long as some of the deeply held convictions are based on religious tenets that make no claim for public reasonableness but merely cite divine command, there are no common available criteria for distinguishing between claims. For instance, the moral tenet underlying a refusal to deliver newspapers that once published allegedly blasphemous cartoons may be as deeply held as the opposition to intentional killing underlying the refusal to perform euthanasia; arguably, moral integrity may be equally at stake in the two cases. A refusal to perform abortion can be grounded in a conception of what vocational (professional) ethics requires; however, so can a refusal to provide intensive care treatment to patients above 70 years of age because they have already received ‘fair innings’ [4]. If we give a right to exemption from abortion and assisted suicide based on moral or vocational integrity, then we should also give a similar right to all others who claim to have deeply held convictions preventing them from performing some of their professional duties. If there were a right to exemption from *any* work task claimed to be against deeply held convictions, this could lead to dramatic consequences for patients, consequences unacceptable in a modern liberal society, which guarantees equal access to public services on the basis of medical need.

Based on these and similar arguments, several recent contributions to the debate reject *any* right to conscientious objection to legal and professionally accepted tasks for any kind of professional. As stated by Schuklenk in an editorial: ‘Societies ought not prioritise individual ideological commitments of some healthcare professionals over patients’ right to receive professional care in a timely and hassle free fashion’ [6], a point he later elaborated with Smalling [7]. However, Schuklenk’s principle in itself does not rule out accommodation as long as patient rights to health services are met.

If the moral integrity and the vocation arguments were indeed the strongest arguments for conscience-based exemption, then the conclusion that conscientious objectors in healthcare should not be accommodated by law would be reasonable. However, we believe that there are strong reasons for a legal right to accommodation in certain cases, reasons that are based on the central role of freedom of conscience in liberal democracies. Recently, Maclure and Dumont have pointed out that the arguments for rejecting freedom of conscience in health professions proposed by Schuklenk and Smalling [7] fail to take into account the broader moral and legal issues concerning freedom of conscience [8]. The arguments of Maclure and Dumont are equally relevant against several others of the main contributors to the bioethical debate on conscientious objection.

Important contributions to the debate have drawn on principles from political philosophy, such as Sulmasy’s analysis of the Lockean concept of tolerance [9] and Trigg’s recent discussion of the ‘principles of liberal democracies’ [10]. However, there has been little discussion of this issue based on a more comprehensive analysis of leading works in political philosophy on the lines suggested by Maclure and Dumont. We address the issue of conscientious objection for health professionals through an analysis of the principle of accommodation of minorities derived from the constitutive values of pluralist liberal democracies. Tolerance is particularly well-founded in questions perceived to involve intentional killing. We will argue that the most fundamental reason for legal toleration of conscientious objection in these cases is the constitutive role of tolerance in liberal democracies under the ‘fact of reasonable pluralism’ [11], and that a circumscribed legal right to accommodation for conscientious objectors is justified by four arguments in conjunction. (1) The need to protect the health professional’s moral integrity. (2) The fact that we may be mistaken in moral judgments, even when we are convinced that we know the truth. (3) The right of minorities to live according to their deeply held convictions, following from basic moral principles of liberal democracies. (4) The special moral and political significance of taking lives. The three first arguments support a right to be heard and to be accommodated as a moral courtesy. Only the last can give basis for a circumscribed legal right to accommodation.

## Main text

### The constitutive moral values of democracy

Liberal democracy is the government of free and equal citizens. In a state of this kind, it is accepted that people should be free to live according to their own conception of the good as long as this does not interfere with other citizens’ right to similar freedom. Each citizen has his or her own ideas of the meaning of life and of what is of value. These conceptions John Rawls calls ‘comprehensive doctrines’. Rawls states that it is a ‘fact that a plurality of conflicting reasonable comprehensive doctrines, religious, philosophical, and moral, is the normal result of [democracy’s] culture of free institutions’ [11]. Conflicts between different ‘comprehensive views’ are not of a character that can be solved by evidence. They are rather perspectives on the evidence that we have in matters that are of moral import. This political recognition of fundamental moral ignorance is the basis for tolerance in liberal democracies. How should the pluralism be handled? Rawls goes on to ‘propose that in public reason comprehensive doctrines of truth or right be replaced by an idea of the politically reasonable addressed to citizens as citizens’ [11]. By adhering to an idea of the politically reasonable we may determine the moral and political values for a society of free and equal citizens who do not necessarily share fundamental conceptions of the good. Some have criticized the Rawls-ian concept of the reasonable for being vague, while others defend it [12]. We hold that the criteria stated below are sufficient to operationalise the concept in the context of conscientious objection.

In this article, we will define reasonable moral and political positions and arguments as those that respect everybody’s reciprocal freedom to think and act according to their own conception of the good, under the requirement that *political* arguments must be based on reasons that everybody can understand and evaluate, even if they do not agree. Liberal democracies must be secular in the sense that religious notions of the good should not determine political institutions, laws and decisions, but this must be a minimal form of secularity, expressed in the idea of an overlapping consensus. In this context, the broader notion of secular values often associated with ‘secularism’, belongs to a *comprehensive* nonreligious doctrine [11]. Thus, reasonable arguments in the context of public reason must be secular in the minimal sense because the principle that arguments must be understandable for everybody requires that no comprehensive doctrine should be privileged, be it religious or secular. They cannot be based on premises derived from comprehensive doctrines such as divine command, meaning that they must be translated into arguments that also those who do not share this fundamental worldview can understand [13]. In addition, they cannot be contrary to scientific facts. A further requirement is that they cannot contradict the equality and freedom of all citizens which is a fundamental tenet of society. This is in keeping with Sulmasy’s requirement that the principle of tolerance should not be used to protect intolerance, i.e. invidiously discriminatory practices [9]. We leave this account of reasonableness as merely a sketch (and also set aside the question of how invidious discrimination should be defined), as the main point of our article lies elsewhere. For more extensive discussions, see e.g. Card [14, 15] and Marsh [16].

There is one of the requirements for reasonableness that needs expansion, as many, perhaps most, conscientious objectors base their refusal on a comprehensive doctrine, often a religious one. If the requirement that their reasons must be translated into sharable premises implies that they must be dishonest, then this is not a good strategy when the aim is to protect moral integrity. However, the demand for translation does not mean that one should deny the real source of one’s objection. It is a demand that one should recognize that in order for others to accept extra burdens, they need reasons that are meaningful to them, even if they do not share the worldview that is the ultimate reason for accommodation. Translating your reasons to something understandable for the one who is accommodating you, is displaying willingness to meet halfway and a recognition of the need for reciprocal respect that is the basis for a well-functioning pluralistic liberal democracy [13].

As the reciprocal freedom of each citizen to live according to their own conception of the good is fundamental for this conception of liberal democracy, ‘the principles of toleration and liberty of conscience must have an essential place in any constitutional democratic conception. They lay down the fundamental basis to be accepted by all citizens as fair and regulative between doctrines’ [11]. Toleration and freedom of conscience, then, are the liberal response to societal pluralism. This ties in with historical debates on the freedoms of religion, speech and conscience as preconditions for the establishment of the modern conception of democracy, as described by Sorabji [17]. We cannot *force* anyone to believe something she or he does not believe even if we can convince or persuade people to change beliefs or force them to express adherence to something they do not believe. Furthermore, we cannot *know* which worldview is the correct one even if we are convinced about the truth of our own. It follows that we should tolerate other views and allow people to act according to their conscience within the limits of the Harm Principle. Due to the fact of pluralism, these have become fundamental moral tenets of liberal democracies.

Maclure and Taylor argue that a liberal democracy that adheres to an open, non-comprehensive secularity as described by Rawls must sometimes make special arrangements for cultural – religious or non-religious – minorities in the public sphere. This is because minorities encounter special challenges in living according to their conscience because their fundamental moral values differ from the values that the majority express through political governance. Indeed, ‘certain public norms applying to all citizens are not neutral or impartial from a cultural or religious point of view’ [18]. Majority values are typically expressed in public calendars, dress codes, language, food customs and some public services – such as the health services. Some of these customs, practices or laws prevent minority citizens from equal opportunity of exercising their freedom to live according to their own conception of a good life.

There is nothing wrong in this. Every society has laws and practices based in their particular cultural history. But in order to ensure equality between citizens regardless of culture and faith, sometimes accommodation for minorities is necessary. One such example is the arrangement of an alternative to military service for convinced pacifists; another is that some countries ensure by law that employees who are not members of the majority faith have a right to leave from work or school in connection with some religious holidays. One may suspect that this is a principle that only gives preferential treatment for religious citizens, but that is not the case; for instance, as pointed out by Maclure and Taylor, an imprisoned vegetarian has a right to vegetarian meals irrespective of the basis for their vegetarianism being secular or religious.

In this context, ‘minority’ is a political, not a numerical, term. A minority is a group of people whose expression of fundamental values is restricted by the laws of the country. Turkey had a Muslim majority during the 1990’s when the use of headscarves was banned in universities, hospitals and other public places. Muslim women who held wearing headscarves in public to be a fundamental religious duty, belonged to a minority in this political sense.

Some do not agree that equal treatment requires accommodation for moral minorities. According to Brian Barry, it is sufficient that the laws and institutions ensure *formal* equality and freedom. If a person’s worldview, be it religious or not, prevents her from exercising that freedom to the same degree as other citizens, this is irrelevant, as she is equal in opportunity with the majority, formally speaking [19]. In essence, however, this amounts to making comprehensive secularism the basis for society, and may make it excessively difficult for minorities to participate in society on an equal footing with the majority.

### Freedom of conscience in professions

It is not clear that the principles of toleration and freedom of conscience can justify tolerance of conscientious objection in professional life. Arguably, changing jobs or even profession is not something that affects fundamental issues of identity, and is a reasonable alternative if the job requires acts that are contrary to conscience. In principle, people choose their own profession, given that they have the required skill and knowledge. They can therefore avoid careers they assume will bring them into conflicts of conscience. If the nature of the work changes (due to, e.g., technological breakthroughs or policy changes), finding a different line of work, either within the same profession or in other fields would often be the reasonable course of action. Particularly in the case of physicians, we are looking at an already privileged group who usually have the opportunity to find alternative employment rather than acting contrary to their conscience. This point about the choice of profession being voluntary is forcefully made by Schuklenk and elaborated further by Schuklenk and Smalling. They point out that many more people want to become doctors than can be admitted to medical school, and that health professionals have a virtual monopoly on their services. They find it ‘reasonable to suggest that doctors refusing to provide professional services that are within the scope of practice should be replaced by someone who is willing to undertake the work’ [7].

However, there is another, weightier reason for toleration of conscientious refusals in the workplace. People have a right to free choice of employment, as stated in the Universal Declaration of Human Rights §23, and any limitation of that right must be based on strong reasons. The same follows from the principles of tolerance and freedom of conscience constituting the moral foundations of a liberal democracy. The default position is that you should not have to choose between your right to a free choice of profession and your fundamental moral convictions. This does not entail that employees are free to retain their work if they refuse to do essential tasks, but there should be strong reasons to force people to make a choice between these fundamental rights [8]. This means that in the context of minority rights we are no longer considering vocation-based conscience specific to health professionals, but instead granting that *anybody in any profession* might encounter a conflict between professional duties and conscience, and that this should as a general rule be avoided through exemptions.

Perhaps this argument is too strong. Many employees in many professions have duties that they find morally problematic. It is practically impossible to give all of them a right to refuse to perform services required by their freely chosen line of work just because they claim to have moral qualms. Such an understanding of a right to conscientious objection would undermine the liberal democracy, as people would not get services they had a right to, and this again could undermine respect for freedom of conscience. In addition, if people had a right to refuse *any* task, referring to their conscience, this could become a way to avoid unwanted tasks for reasons of mere convenience. In order to avoid that, any right to accommodation must be conditional and restricted.

A general legal right to accommodation should not be enshrined in law, because it would grant accommodation on grounds that fail to fulfill the demand of reasonability, and also when accommodation would entail harm to significant competing goods. A secular citizen can understand that a Muslim refuses to drink alcohol or eat pork due to a divine prohibition, but still hold it unreasonable that this should be extended to a refusal to deliver bottles or packages containing these goods to non-believers in cases where this would put extra burdens on customers, colleagues or employer. Several cases of conscientious objection are like this: someone will have to carry a burden for a conviction that they cannot regard as reasonable, because it is based in a worldview they do not share. Thus, there should not be an *unconditional* right to such refusal in a liberal democracy, as it would unduly infringe on the corresponding right of other citizens.

For there to be a legal right to conscientious objection, it must be limited to cases where the moral basis is more fundamental in a sense that all reasonable citizens should accept – even when they disagree, in accordance with the ideals of public reason. They must be presented in a minimally secular frame, in accordance with scientific facts and non-discriminatory. We will argue that objections to performing what is perceived as intentional killing satisfies these requirements. This does not mean that these cases are the only instances that can be protected by law. There may be specific instances of conscientious objection that are protected by law in some jurisdictions due to particular historical reasons or as a result of specific political compromises. We do not hold that this is wrong. Our argument is restricted to the claim that conscientious objection to perceived intentional killing ought to be protected by law in any pluralistic liberal democracy. In order to show that, we must establish the special status of taking human life in this kind of society, as illustrated by the status of conscientious objection to active military service.

### The special moral status of intentional killing

The fact that there is a legal right to conscience-based exemption from military service indicates that liberal democracies' citizens value freedom of conscience and that this protection is considered to be particularly warranted in matters of life and death. The roots of the idea that there should be a conscience-based exemption from military service is directly connected to the rise of the ideas of liberal democracy [17]. The main arguments for this freedom of conscience occur repeatedly throughout its philosophical history: First, it is *ineffective* to try to force people to act contrary to their fundamental convictions, because that will only lead to hypocrisy, not a sincere change of mind. This will negatively affect their behaviour as they will be unwilling soldiers, in the worst case undermining the combat strength of the army and putting their fellow soldiers at risk. This argument does not imply that they should be exempt from non-combat service in the army, only that they should be exempt from killing other people. Second, in (at least some) religious and moral questions we are in a state of *ignorance* as we have no way to know that our beliefs are the truth and that those who disagree with them err. Some “know” that lying is always wrong because they are orthodox Kantians, while others “know” that it is often right to lie to protect others from suffering based on a utilitarian conviction. Even if both parties think they know the truth, at least one of them is wrong and there is no way to prove which conviction is the truth. Forcing others to act according to *our* convictions, then, may lead them to turn from truth to error [17]. We can add that preventing people from acting in accordance with their conviction is particularly bad if the action has existential significance by concerning their fundamental moral worldview.

*Respecting* my opponents’ differing judgement does not imply *acceptance of a moral mistake*, as Giubilini assumes [20], although he may be right in his claim that moral integrity alone is not sufficient to justify such respect. Rather, the respect is an expression of the idea of tolerance as a basic value in a liberal democracy under the fact of reasonable pluralism. This is a political conception of tolerance, not a moral one, which is the target of Giubilini’s criticism. Conflicts between different belief systems, moral worldviews, or what Rawls calls ‘comprehensive views’, are not of a character that can be solved by evidence. They are rather perspectives on the evidence that we have in matters that are of moral import. As we have seen above, this political recognition of fundamental moral ignorance is the basis for tolerance in liberal democracies.

Freedom of conscience to refuse military service became highly relevant in the UK during the First World War. The state recognised a plurality of reasons for opposing military service; tribunals exempted men on ‘religious, *moral*, or *socialistic* grounds of political liberty’ [17]. (At that time, significant parts of the socialist movement were anti-militaristic, holding that war was an integral part of capitalism.) Although most people believed these objectors to be morally wrong, they supported the exemption because they accepted that it would be wrong to force someone to act against conscience in the case of military service. They recognised ‘that those who were sincere would also be wrong to go against their consciences’ [17]. This implies a distinction between what they think is a substantial moral mistake on the part of the objector and a principle that it is morally wrong for *anybody* to act against conscience, which is established in the Western tradition. In order to ensure that only convinced pacifists were accommodated, objectors had to justify their refusal to serve for a military tribunal and do alternative work, which often was of an unattractive nature.

Objection to military service has a paradigmatic role in modern debates on conscientious objection due to the underlying widely shared moral view that it is wrong to kill human beings. Although most hold that there are circumstances that warrant such killing, such as self-defence and war, as is evident from the general support for the army, most would also find it *reasonable* to hold that it is wrong even in such circumstances. There is a moral seriousness to taking life that makes wholesale rejection of intentional killing a moral stance deserving of respect; thus it is acceptable that some would object to military service or to participating in executions. Furthermore, pacifism – justified in many different ways – is an established tradition both in Western and other cultures, making it more than a marginal view connected to particular sects or local circumstances. Pacifism is a reasonable position to take, because it is grounded in reasons that are widely understood, even by people who disagree strongly on the conclusion. Therefore, the argument from moral ignorance supports toleration of the conscientious objector.

The historical arguments for conscience-based exemption from military service show that there is a precedent for tolerating refusals to act contrary to conviction in matters that the objector perceives to be of existential significance, for two reasons. First, else they would not act with conviction, which means they would do a bad job. Translated to modern discussions in the health service context, this argument appears to be an aspect of the *moral integrity argument*. Second, in some contested issues we cannot know what is morally right, even if we know that there is such a thing as moral rightness. This is the *argument from moral ignorance.* In the political context of liberal democracies, these two conditions – a threat to moral integrity and the absence of proof that the objector is mistaken – are necessary conditions for there to be a right to conscience-based exemption. Furthermore, with the establishment of modern democracies, objections to killing people were seen to have a special moral status; one should never be forced to kill in situations where one is convinced this killing is morally wrong.

### Legal right to exemption from killing

Although exemption from military service is reasonable in a liberal democracy, based on the arguments above, it does not follow that there should be an analogous exemption from the performance of particular tasks for health professionals. The first is a compulsory service; the second is part of a voluntary choice. What the military service case does is merely to establish the fundamental status in liberal democracies of conscientious objections to killing. We furthermore agree with Maclure and Dumont that weighty reasons are required to force citizens to choose between their basic rights to free choice of profession and to freedom of conscience. Based on this, we will now argue that there are cases where there is *a right to conscientious objection* and where this right should be *a legal right*. These are the cases where some consider the act as the intentional taking of human life, based on reasonable arguments. The most obvious cases are abortion and assisted dying. The question of whether this right should include objections to *refer* for services that arguably involve taking human lives, depends on the question of the moral character of such referrals. This issue has been discussed extensively in the literature and depends, e.g., on theories of moral complicity in evildoing; we elect not to enter into this discussion here.

We have presented four main arguments that together support a legal right to exemption from procedures that involve intentional killing: moral integrity, the fact of moral ignorance in some hard cases, equality for moral minorities in liberal democracies and the special status of taking human life. We now briefly discuss three ancillary arguments that support our stance and help define and justify its scope: 1) the generally accepted unique worth of human life, 2) the tradition for accepting reasoned conscientious refusals of killing, and 3) the long-ranging and deep moral controversy in society concerning several issues that involve taking lives.

#### The unique worth of humans

Moral theories and cultural traditions typically accord special value to human life. Especially influential on Western culture has been Kant’s line of reasoning. Stating that everything may be given a certain value, Kant stressed that only human beings have *worth*, which means incomparable value. This Kant expressed by saying that every human being is irreplaceable and ought to be treated as an end in itself, never only as a means [21]. Taylor points out that this is a modern notion, usually expressed in the concept of “inherent dignity” that encompasses every human being in an egalitarian sense [22]. Sulmasy describes how it is fundamental in human rights thinking, and important in ethics in general [23]. Such notions of the unique value of every person are generally accepted as the basis for everybody’s freedom and equality in liberal democracies, even if one does not accept Kant’s philosophical system in full [24]. The purpose of society is to ensure the freedom and equality of each and every citizen. One reasonable interpretation of this principle is that it is morally wrong to intentionally end the life of any person, as this is an ultimate end to their freedom. This is not just *any* moral wrong, but a fundamental principle for a liberal democracy.

However, this invites the further question of the principle’s limits. Does the principle that it is morally wrong to end a person’s life extend to the unborn and to, e.g., people with severe degrees of dementia or in a persistent vegetative state? Are all human beings also human persons in the moral sense? It is not necessary to rehearse the well-trodden paths of such debates here, only to point out that there are several positions here that are *reasonable*, in the sense stated above, i.e. that they are not based on religious or other comprehensive principles, do not contradict scientific facts and are non-discriminatory. This includes also the most inclusive position, which claims that all that are human beings in a biological sense are also human persons morally speaking, thus including also human foetuses, embryos and even zygotes within the ambit of morally valuable human lives worthy of protection. There is an extensive literature supporting this position with nonreligious arguments [25–27], and one does not have to think that it is a convincing position in order to accept that it is reasonable. Many who object to perform abortion do so on religious grounds. Accepting that they have to translate these religious arguments to nonreligious, for example the ones referred above, does not imply that they discard the fundamental motivation for their objection. It is merely a way of displaying respect for those that accommodate them by seeking to make their argument understandable without requiring the acceptance of contested metaphysical assumptions. If they are unwilling to attempt such translation or their attempt fails, we hold that the conditions for a legal right to accommodation is not fulfilled.

#### The right to conscientious objection to taking life

Even if everybody agrees that it is wrong to kill humans, there are arguable exceptions to this general principle: taking life in legitimate war, in capital punishment, and in assistance in suicide at the person’s own request. The two latter exceptions are legal in a limited number of democracies, but all three have in common that they are forms of killing people that are legal in one or several liberal democracies. Some hold that it is wrong to take life even when it is sanctioned by the state, and, as discussed, in most liberal democracies pacifists have a right to exemption from military service. We saw above that this is a strong tradition in the Western world, and closely connected to the awareness of the fundamental value of tolerance under conditions of pluralism. Being forced to take lives when one judges it to be wrong is particularly problematic from a moral point of view, due to the fundamental role the respect for human life has for our political system. Even if we disagree about what constitutes illegitimate killing, we should agree on the fundamental role that respect for human life plays in most people’s moral outlook, and accept that we interpret the scope of legitimate killing differently.

Should tolerance also extend to refusals to perform abortion and euthanasia? Euthanasia is not a case of killing someone who wants to continue life, as is the regular case in war and capital punishment. If the basic value of humanity is expressed in everyone’s freedom, understood as the right to self-determination, then this killing is basically different from the paradigmatic cases of war and capital punishment. One is helping someone carry out her freely chosen act. However, this is not the only reasonable interpretation of the idea of the fundamental value of human life. One can argue that our fundamental freedom of self-determination does not include the freedom to end our lives and thereby any further exercise of self-determination. On this view, it is wrong not only for moral reasons, but also for political reasons – just as it is morally and politically wrong to sell oneself as a slave. This follows for example from a Kantian interpretation of the principle of freedom as autonomy [28]. The fact that conditions for lawful assisted dying are set out in detail also indicates that this is not merely a question of helping someone who wants to exercise their freedom by ending their life. It is reasonable, in the sense stated above, to consider such assisted suicide to be illegitimate killing, and citizens should therefore have a legal right to be exempted from performing such acts if they hold them to be contrary to their conscience, without this infringing on their right to freely choose jobs. One way to solve this is through the Dutch system where assisted dying is not a patient right and doctors are free to refuse any involvement; thus there is no need for exemptions for conscientious objectors.

Abortion is a different case, as this is clearly not a choice by the one whose life is terminated. The question is whether it is reasonable to consider this the killing of a person. The general agreement that it is wrong to kill human beings, combined with the reasonable disagreement concerning the scope of human personhood, makes moral opposition to the killing of foetuses, embryos and zygotes a reasonable position to hold. As long as we agree that people should have a legal right to be exempt from killing human beings, one should have a right to be exempt from abortion if one regards the foetus as a person.

An interesting case is conscientious objections to inserting intrauterine devices (IUDs) for contraception, where such objections are grounded in the belief that the IUD can act as an abortifacient. This case illustrates important points about the relationship between moral opinions and facts, and degrees of moral complicity, respectively. Firstly, in order to warrant societal toleration an opposition to a service must not be based on views that are demonstrably factually erroneous. In the case of objections to IUD insertions it then becomes a crucial premise whether IUDs in fact have abortifacient effects. This is a contested issue; abortifacient effects of IUDs are claimed by some reviews [29]. Secondly, even granted that IUDs have abortifacient effects and that the embryo whose life the IUD might end is a human person in the moral sense, the physician’s causal contribution to the ending of innocent life is arguably of a different kind than in military service, executions, abortion and assisted dying. Specifically, if any abortifacient effect occurs this might well be an unintended (and unwanted) effect from the physician’s point of view, whereas in the paradigmatic cases of killing just mentioned, killing is typically intentional, even if one seeks to minimize it as is usually done in war. As is commonly accepted, there is a particular moral *gravitas* to killings that are intentional. Thus, the difference in intention is a morally relevant difference. There are likely to be different views on whether *unintentional* killing should also be included within the ambit of the life-and-death cases in which accommodation should be secured by law.

#### Deep public disagreement

The disagreements over abortion and euthanasia are not ordinary political debates in our democracies. In most countries, the debates concerning these issues have been harsh and divisive, often continuing with equal fervour after policy decisions have been made. Many countries have admittedly settled the issue through a relatively stable *modus vivendi*, often including accommodation for conscientious objectors. Still, the controversies indicate that these moral issues are considered to be of special significance for many of the participants in the debate, that it is difficult to accept the result, and that no parties to the debate can *know* the veracity of their stance, even if they claim to do so. In each case there is a political minority that has lost a political struggle thought to be of vital importance. Thus, they have to live with a political decision they think is fundamentally morally wrong. In such a situation, the majority, who have won the struggle, must still respect the rights of the ‘moral minority’; indeed, they might want to reach out in reconciliation to those who have lost. This can be achieved by appreciating the losing part’s continued moral problems in living with laws they find to be fundamentally wrong, including not forcing them to choose between taking part in the performance of these legal activities or losing their job.

Regarded in isolation, this last principle could lead to a demand for accommodation in a number of divisive issues. Our point is merely that this last argument, in conjunction with the previous two, strengthens the special status of legal procedures considered by some to be illegitimate, intentional killing of human beings. The divisive nature demonstrates that such issues have special significance compared to other matters of conscience, and should be treated differently.

### Qualifications to the right to accommodation

We have argued that if the health professional’s conscientious refusal is built on opposition to partaking in what the professional plausibly takes to be intentional killing, the professional should have a legal right to accommodation. In the case of accommodation as a ‘moral courtesy’, the employer has likely weighed the competing interests and found that accommodation can be granted without significant harms to other interests at stake. The law, however, is a blunt instrument, and a legal right to accommodation means that harm to the interests of patients, colleagues and employers cannot always be avoided; at least, the avoidance of such harm cannot fully be guaranteed beforehand. Acceptable harm to patients does not include restrictions on their right to legal health services, but may include delays and the burden of extra travel [5, 9]. Likewise, the objector’s colleagues may have to cover for them (but should in return be relieved of other tasks they find unattractive). This, we think, are consequences that society should be willing to accept because of the reasons in favour of such legal conscience clauses. However, it is also an important argument for equipping conscience clauses with justified qualifications.

Such qualifications must include the requirement to provide a reasonable account of the basis for objection, and cooperation in reducing burdens to patients and colleagues [5]. There may also be a limit to how many professionals may object to participating in the controversial task. One example of this kind of qualification is found in the Norwegian regulation of abortion. If the number of objectors in the clinic exceeds what is required for securing patient access, the management may refuse to employ further conscientious objectors. In this way, one could for instance avoid the obstacles facing women seeking legal abortion in Italy [30].

Our argument thus supports a *legal* right to accommodation for objectors in cases that involve what can reasonably be perceived as killing. It does not imply that objection to other tasks is necessarily less serious or less worthy of protection from a *moral* point of view. In practice, the demands on the objector are similar in the two cases: reasonable justification and willingness to compensate the burdens inflicted on others. Likewise, the expected effect on others is a constraint on accommodation in both cases. The main difference is that when there is a legal right, it will not be up the employer to decide whether conditions for exemption are fulfilled, but ultimately to courts of law. This strengthens the protection of the objector considerably, in that toleration becomes less dependent on the employer’s judgement and goodwill. Protection of moral integrity is important to secure an inclusive work life in a pluralistic society, but it is only in exceptional cases that this should be subject to legal protection. These must be connected to fundamental values of liberal democracies, such as the inviolability of human life.

## Conclusion

There are strong reasons for a limited accommodation for conscientious objectors in the health professions in liberal democracies. This should take two forms: a legal right to refuse reserved for cases of participation in what can be reasonably considered as intentional killing; and as a moral courtesy in other cases where moral integrity is at stake when accommodation does not violate third party, colleague and employer interests. In this paper, we have argued that a legal right is justified by four arguments in conjunction: the need to protect the health professional’s moral integrity; the fact that we may be mistaken in moral judgments; the right of minorities to live according to their deeply held convictions, following from basic moral principles of liberal democracies; and the special moral significance of taking lives.
